# Effect of magnetic fullerene on magnetization reversal created at the Fe/C_60_ interface

**DOI:** 10.1038/s41598-018-23864-8

**Published:** 2018-04-03

**Authors:** Srijani Mallik, Stefan Mattauch, Manas Kumar Dalai, Thomas Brückel, Subhankar Bedanta

**Affiliations:** 1 Laboratory for Nanomagnetism and Magnetic Materials (LNMM), School of Physical Sciences, National Institute of Science Education and Research (NISER), HBNI, Jatni, 752050 India; 2 Jülich Centre for Neutron Science (JCNS), Heinz Maier-Leibnitz Zentrum (MLZ), Forschungszentrum Jülich GmbH, Lichtenbergstr. 1, 85748 Garching, Germany; 3 CSIR - National Physical Laboratory, Dr. K. S. Krishnan Marg, New Delhi, 110012 India; 4 Academy of Scientific and Innovative Research (AcSIR), CSIR-National Physical Laboratory, New Delhi, 110012 India; 5 CSIR - Institute of Minerals and Materials Technology, Bhubaneswar, Odisha, 51013 India; 6 PGI-4: Scattering Methods Forschungszentrum Jülich GmbH, 52425 Jülich, Germany

## Abstract

Probing the hybridized magnetic interface between organic semiconductor (OSC) and ferromagnetic (FM) layers has drawn significant attention in recent years because of their potential in spintronic applications. Recent studies demonstrate various aspects of organic spintronics such as magnetoresistance, induced interface moment etc. However, not much work has been performed to investigate the implications of such OSC/FM interfaces on the magnetization reversal and domain structure which are the utmost requirements for any applications. Here, we show that non-magnetic Fullerene can obtain non-negligible magnetic moment at the interface of Fe(15 nm)/C_60_(40 nm) bilayer. This leads to substantial effect on both the magnetic domain structure as well as the magnetization reversal when compared to a single layer of Fe(15 nm). This is corroborated by the polarized neutron reflectivity (PNR) data which indicates presence of hybridization at the interface by the reduction of magnetic moment in Fe. Afterwards, upto 1.9 nm of C_60_ near the interface exhibits magnetic moment. From the PNR measurements it was found that the magnetic C_60_ layer prefers to be aligned anti-parallel with the Fe layer at the remanant state. The later observation has been confirmed by domain imaging via magneto-optic Kerr microscopy.

## Introduction

Organic spintronics is an emerging field in 21^*st*^ century both from fundamental research and application point of view^1,2^. The major aspects in OSC/FM multilayers such as spin injection, tunneling magnetoresistance (TMR), giant magnetoresistance (GMR), hybrid interface properties are observed recently^3–6^. OSCs are favorable candidates for information processing technologies due to their constituents low weight materials (e.g. Carbon, Hydrogen etc.) having weak spin orbit coupling (SOC) and hyperfine interactions^1,2^. Therefore, OSCs exhibit comparatively longer spin relaxation and dephasing times (>1 *μ*_*s*_) than the conventional inorganic semiconductors^7^. Along with these, the flexibility, low cost production, and versatility in synthesis of OSCs are desirable for the devising and spintronics applications.

Among various available OSCs, Buckminsterfullerene (C_60_) has drawn immense research interest in organic spintronics because it exhibits the following properties viz. low spin orbit coupling (although in liteature it has been shown that curvature induced SOC can be present)^8,9^, large spin diffusion length at room temperature, thermal and mechanical resilience, generation of transient triplet state and required mobility to be used in organic field effect transistors (OFETs)^10–12^. C_60_ is even more prudent due to the absence of hyperfine interaction as it consists of only C atoms. Spin injection in C_60_ has been studied extensively by recording the magnetoresistance (MR) in spin valve devices with C_60_ as spacer layer^12–14^. A high TMR of ~80% at near zero bias voltage at 2 K has been observed in granular C_60_-Co films^15^. Further, tunneling anisotropic magnetoresistance (TAMR) has been observed in a spin valve structure having only one ferromagnet (Co) as bottom electrode and C_60_ as the spacer layer^16,17^. There are several reports regarding the observation of GMR, TMR, TAMR in C_60_ based spin valve devices^13,15–18^ and various models have been considered to explain the MR behavior^19^. The performance of the OSC based spintronic devices is mainly dependent on the OSC/FM interface properties^20,21^. There are two major aspects which need to be addressed in such systems having OSC/FM hybrid interface. First is the charge transfer phenomena at the hybrid interface which leads to induction and reduction of magnetic moment in the OSC and FM layers, respectively. The second aspect is to understand the effect of the magnetic hybridized OSC layer on the magnetic properties viz. magnetization reversal mechanism, anisotropy symmetry, domain structure etc. of the FM/OSC system. There are a few reports where the aforementioned first aspect has been discussed and there the hybridized interface has been termed as ‘spinterface’^22^. For example, in Co/C_60_ multilayers, an induced moment of 1.2 *μ*_*B*_ per cage of C_60_ has been observed at the cost of ~21% suppression of FM moment in the Co layers^23^. Polarized neutron reflectivity and x-ray magnetic circular dichroism measurements have revealed the presence of an antiferromagnetic coupling between the interfacial layers of cobalt and C_60_^23^. Similarly, the hybrid interface between Fe and C_60_ leads to magnetic moments of the C_60_ to *μ*_*S*_ = −0.21 and −0.27 *μ*_*B*_ per molecule where it was aligned antiparallel on Fe(001) substrate and Fe on W(001) substrate, respectively^24,25^. However, in literature the second aspect i.e. the effect of hybridization on the magnetic properties of the FM layer has been rarely discussed. Recently, Bairagi *et al*. have shown that the presence of a C_60_ layer on Co ultrathin film can control the anisotropy symmetry of the Co layer^26^. An inverse spin reorientation transition from in-plane to out-of-plane was observed due to the local hybridization between C_60_ p_*z*_ and Co ${{\rm{d}}}_{{z}^{2}}$ orbitals^26^. In this context, it is very important to understand the effect of such magnetic interface in FM/OSC layers on the magnetization reversal which is very much necessary for applications of these heterostructures in any spintronic devices.

In this paper, we have studied the magnetic interface in bilayer of epitaxial Fe/C_60_ by polarized neutron reflectivity (PNR) and quantified the induced magnetic moment in the organic layer. Further we studied the magnetization reversal process in such bilayers and compare it with the single Fe film to elucidate the effect of the magnetic interface on the domain structure.

## Results and Discussion

**Figure 1:**
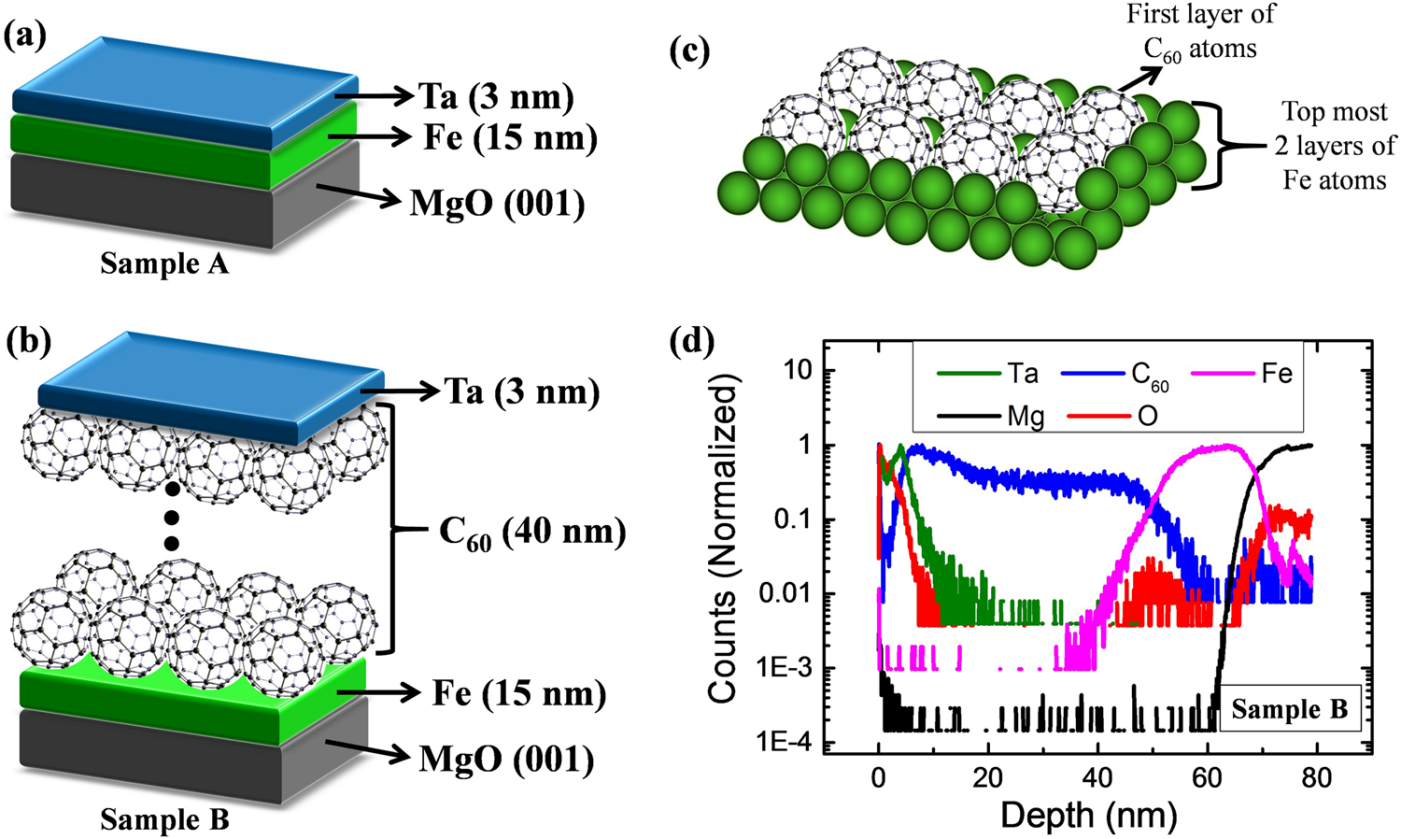
Schematic layer structure for (**a**) sample A and (**b**) sample B. (**c**) Schematic structure of the Fe/C_60_ interface where the C_60_ cages are embedded in the top most Fe layer due to the surface reconstruction. (**d**) Depth profile of sample B measured using secondary ion mass spectroscopy (SIMS) technique.

Figure 1(a,b) show the schematic of the layer structures in samples A and B. It has been reported that C_60_ adsorption on Fe surface leads to surface reconstruction when the deposition temperature is in the temperature range of 70 °C to 300 °C^24,27^. Yang *et al*. have performed ab initio calculation to show that for Fe/C_60_ interface, 4 atom hole reconstruction is the most stable adsorption structure where a pentagon of the C_60_ cage sinks-in a 4-atom hole of top most Fe atomic layer^27^. In this context it should be noted that the Fe/C_60_ bilayer (sample B) studied in this paper is prepared at 150 °C. Therefore, similar mechanism of four-atom hole reconstruction might be possible in sample B. Figure 1(c) shows a schematic of such a reconstructed Fe/C_60_ interface. However, depending on the roughness at the interface, intermixing of Fe and C_60_ is also quite probable.

To understand the nature of the interface between the layers, TOF-SIMS has been performed on sample B. Figure 1(d) shows the depth profile for the layer structure of sample B obtained from the SIMS measurement. The rising and falling of different colors in the figure represents the appearance of the respective layers with respect to the depth from the surface of the sample. The depth profile data clearly shows the multilayer structure of MgO (001)/Fe(15 nm)/C_60_(40 nm)/Ta(3 nm) (Sample B) accordingly as per the proper growth structure. The high resolution TOF-SIMS^28,29^ clearly ressolves the individual layers of the sample. The profile starts with high intensity of Ta (Green) along with O (Red) represents the top most layer and it starts decreasing when the intensity of C_60_ (Blue) rises up. The C_60_ layer continues till the next layer of Fe (Pink) come up. The Fe layer is just above the MgO substrate (Mg is black and O is Red). The thickness of each layer has been determined approximately from Fig. 1(d) as ~14.53 ± 3.34 nm, ~44.58 ± 3.28 nm, ~2.94 ± 1.23 nm for Fe, C_60_ and Ta, respectively. The nature of the interface i.e. the presence of inter-diffusion depends on the position of the intercept between the falling and rising edge of two consecutive layers. It can be seen from Fig. 1(d) that the coordinate of the intercept between all the consecutive layers lie a little above the 50% of the highest intensity. This confirms the presence of very thin layer of inter-diffusion (<1 nm) between all the consecutive layers. However, it was not possible to calculate the exact thicknesses of the inter-diffusion layers as it was beyond the resolution limit of the technique.

**Figure 2:**
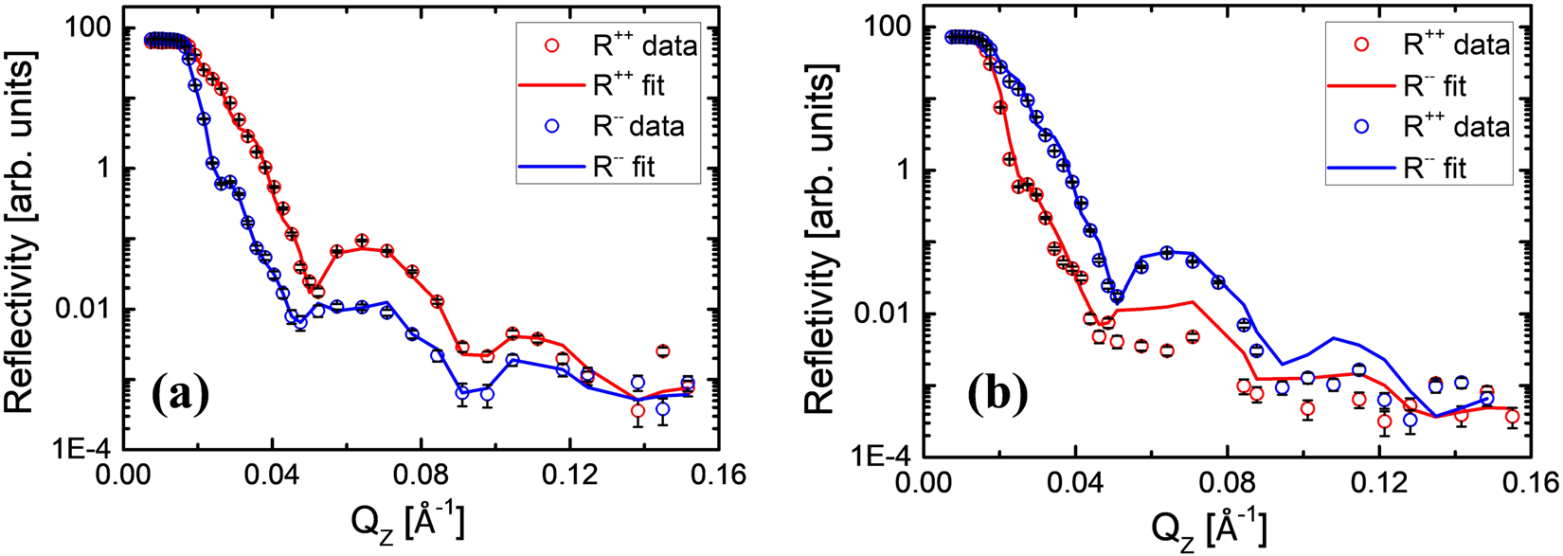
Polarized neutron reflectivity (PNR) data for sample B measured at room temperature at (**a**) saturation (*μ*_0_H = 100 mT) and (**b**) near remanence (*μ*_0_H = 2.1 mT) for magnetic field applied along *ϕ* = 45°. The PNR data (open circles) and their corresponding fits (solid lines) for R^++^ and R^−−^ channels are shown by red and blue color, respectively.

**Figure 3:**
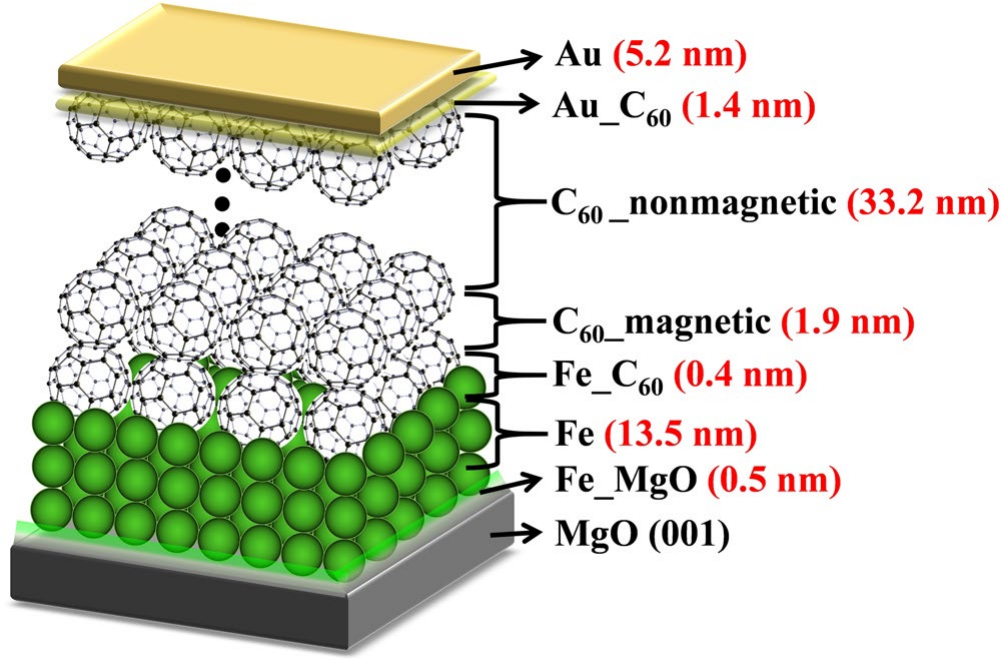
The model layer structure for sample B where the thicknesses for each layers were obtained by fitting the PNR data shown in Fig. 2. Fe_MgO (0.5 nm) and Au_C_60_(1.4 nm) layers correspond to the intermixing layers between Fe & MgO and Au & C_60_, respectively. Fe_C_60_(0.4 nm) is a thin layer which corresponds to the surface reconstructed layer (shown in Fig. 1(c)) at the inteface between Fe and C_60_. C_60__magnetic corresponds to the C_60_ layer which exhibits magnetic moment of 2.95 *μ*_*B*_/cage (as per the best fit). Further, C_60__nonmagnetic corresponds to the remaining C_60_ layer which do not exhibit any magnetic moment.

Neutron is a powerful probe to provide layer selective structural as well as magnetic information in multilayers. Figure 2(a) shows the PNR data measured at saturation (*μ*_0_H = 100 mT) along *ϕ* ~ 45° (cubic hard axis, see the MOKE discussion). The red and blue open circles correspond to the data for up-up and down-down reflected neutron intensity, respectively. Here, the reflectivity for up-up reflectivity is more than that of the down-down case for lower Q values. This confirms that the sample was at its positive magnetized state. The data was fitted using GenX software^30^, which is based on the Parratt formalism^31^. The thickness of the layers of sample B were extracted from the best fits and the values are similar with the ones evaluated from the SIMS data. From the PNR fits we have obtained the magnetic moments for each layer of the sample B. The Fe layer exhibits magnetic moment of 1.59 *μ*_*B*_/atom, which is less by ~27% than its bulk value (2.2 *μ*_*B*_/atom). The PNR fits indicate that a thin layer (0.38 nm) of interdiffusion is present at the interface of Fe and C_60_ layers. This layer probably corresponds to the reconstructed interface where the top most Fe atomic layer and the lower portion of the C_60_ cages of the bottom most C_60_ molecular layers are involved. It should be noted that we have tried to fit for various thickness of the interdiffusion layer. However, the best figure of merit was obtained for the thickness of 0.38 nm. Further, we note that the roughness at the interface is high because of the high temperature growth and large lattice mismatch between Fe and C_60_. Therefore, this interdiffusion layer may be as a result of surface reconstruction or could actually be simple intermixing. The Fe_C_60_ interdiffusion layer exhibits a magnetic moment of 5.14 *μ*_*B*_/unit where 1 unit consists of one Fe atom and one C_60_ cage (60 atoms). On top of this interdiffusion layer there is about 40 nm of pure C_60_ film which is confirmed from the density parameter used in the PNR fitting. However, in this 40 nm of C_60_ layer, upto 1.9 nm of C_60_ near the Fe-Fullerene interface exhibits a magnetic moment of 2.95 *μ*_*B*_/cage (as per the best fit). The reason behind the induced magnetic moment in pure fullerene cages is the hybridization between Fe and C atoms at the interface. C atom has the affinity for electrons according to its electronic structure and Fe can donate electrons from its d-orbital to reach the lower energy configuration^25,27,32^. Due to such polarized charge transfer from Fe to C atoms the fullerene balls exhibit magnetic moment and therefore Fe moment is less than its bulk value. We have tried to fit the PNR data for various values of magnetic moment in the range of 1 to 5 *μ*_*B*_ for the magnetic C_60_ interface. We have found qualitatively similar fits in comparison to Fig. 2(a) when considering C_60_ moment in the range of 1 to 5 *μ*_*B*_. However for *μ*C_60_ = 2.95 *μ*_*B*_/cage the figure of merit was the lowest which indicates that this is the best fit. This can be further confirmed by referring to the Supplementary Fig. S2 showing the figure of merit for various C_60_ moments. We note that the DFT calculation by Moorsom *et al*. have shown that 1.3 e charge transfer between the metallic ferromagnet to the C_60_ interface can yield magnetic moment up to 3 *μ*_*B*_^23^. This implies our obtained moment of 2.95 *μ*_*B*_ is a reasonable moment which does not need to be a result of 3 electron charge transfer. However if we consider that the induced magnetic moment in C_60_ is a direct result of equivalent charge transfer, then 2.95 *μ*_*B*_ magnetic moment is obtained because of ~3 e charge transfer. In such case this leads to a potential of ~2 V which may be unphysical (see supplementary information for detailed calculation). Considering this argument of spin moment directly coupled to equivalent charge transfer the induced magnetic moment in our case might be ~1.5 *μ*_*B*_. Nevertheless the actual phenomena of spin transfer coupled to charge transfer needs to be understood via future theoretical calculations to elucidate if such high moment of ~3 *μ*_*B*_ is possible to be induced in C_60_ interface. Figure 2(b) shows the PNR data for sample B measured near the remanence (*μ*_0_H = 2.1 mT) along *ϕ* ~ 45° (cubic hard axis). We have incorporated the structural parameters used in the best fit for the saturation data (Fig. 2(a)) for fitting the PNR data measured at remanence (Fig. 2(b)). It is found that near the remanence about 88% of the Fe spins have reversed in comparison to the saturation state. However, the magnetic C_60_ layer exhibits a positive magnetic moment of 2.95 *μ*_*B*_/cage in the vicinity of the interface (~0.2 nm). However, next to the interface (~1.7 nm) induced moment in the magnetic C_60_ becomes weak. It should be noted that, the fullerene layer is antiferromagnetically coupled to the Fe layer at the remanence state. The layer structure and the thicknesses of each layers obtained from the PNR fit is depicted in Fig. 3.

**Figure 4:**
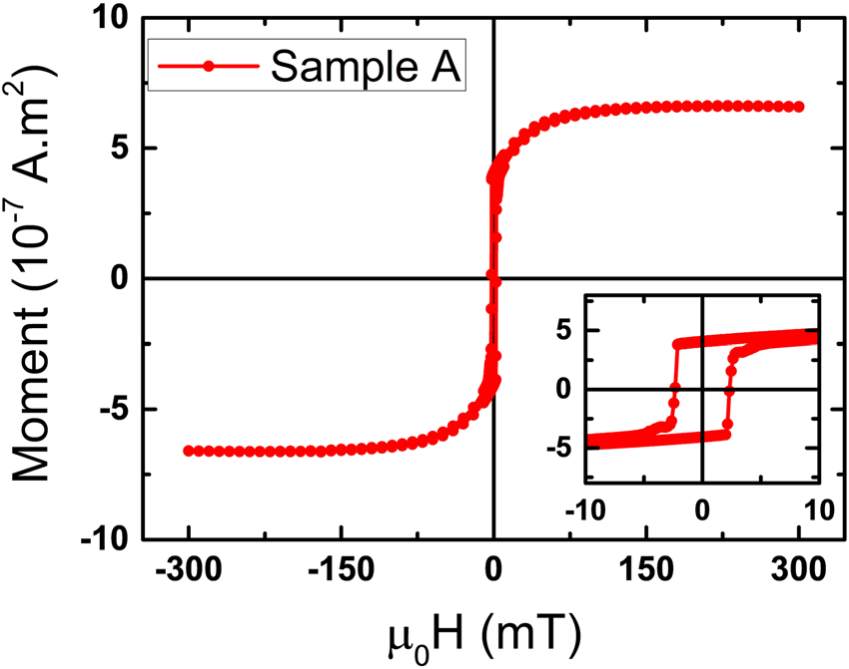
Hysteresis loop for sample A measured along the cubic hard axis i. e. *ϕ* ~ 45° at room temperature using superconducting quantum interference device (SQUID) magnetometry to calculate the magnetic moment of Fe in sample A. The inset of the figure shows the zoomed version of the hysteresis loop near the coercivity.

In order to calculate the magnetic moment in sample A, hysteresis measurement (Fig. 4) was performed using superconducting quantum interference device (SQUID) at room temperature. By measuring the volume of the sample and the saturation magnetic moment we have obtained the magnetic moment in sample A to be 2.26 *μ*_*B*_/atom, which is comparable with the bulk magnetic moment of Fe. This analysis confirms the PNR analysis for sample B that there is a reduction (~27%) in magnetic moment in the Fe layer as compared to the control sample A.

**Figure 5:**
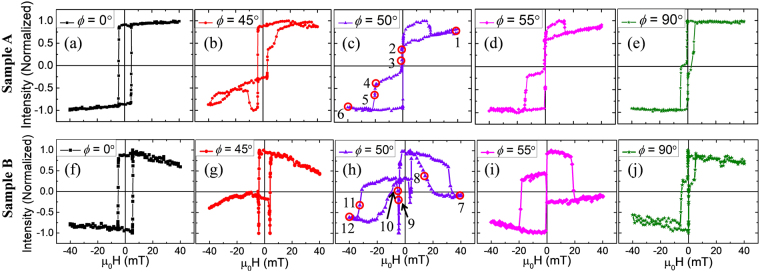
Hysteresis loops measured by longitudinal MOKE magnetometry along different angles (*ϕ*) between easy axis and applied magnetic field. The hysteresis loops shown in (**a**–**e**) correspond to the single layer of Fe thin film i.e. sample A. Similarly, the hysteresis loops shown in (**f**–**j**) correspond to the Fe/C_60_ bilayer thin film i.e. sample B.

From the PNR studies it is confirmed that there is a thin layer (~2 nm) of magnetic C_60_. It is desirable to study the magnetization reversal in such ferromagnetic/organic interfaces. In this context we have performed MOKE magnetometry on both the samples. Hysteresis loops were measured by varying the angle (*ϕ*) between the magnetic field direction to the easy axis of the samples. Figure 4 shows the hysteresis loops for both the samples A (Fig. 4(a–e)) and B (Fig. 4(f–j)). For different *ϕ*, one or two stepped hysteresis loops were observed in both the samples. Such shape of the loops can be explained from the anisotropy nature of the samples. It is known that Fe follows epitaxial growth when deposited with specific preparation conditions on MgO (001) substrate^33,34^. The epitaxial relation between bcc Fe and MgO is Fe(001)[110]$\parallel$MgO(001)[100]^35^ and this leads to cubic anisotropy. It should be noted that due to the geometry of our deposition chamber the Fe film (for both samples A and B) was deposited under oblique angle of incidence. Systematic studies by many groups have revealed that depositing magnetic films under such oblique incidence leads to uniaxial anisotropy^33,36–38^. In our previous study on a similar Fe film with thickness of 25 nm, we found that the easy axes of both the anisotropies are superimposed with each other^33^. The uniaxial and cubic anisotropies follow *π*/2 and *π*/4 symmetries, respectively. Therefore, one of the easy axes of the cubic anisotropy lies parallel to the hard axis of the uniaxial anisotropy. In samples A and B studied in this paper, the anisotropy symmetry is very similar. A schematic showing the anisotropy configuration of the samples are depicted in Fig. S3 of the supplementary information. Figure 5(a) show the hysteresis loop for sample A along the *ϕ* = 0° i.e. easy direction for both uniaxial and cubic anisotropies. Therefore, this axis is the lowest energy direction and magnetization reversal occurs via two simultaneous 90° domain wall (DW) motion^33^. In the positive saturation state all the spins point along [100] direction. By decreasing the field towards negative side, the spins flip towards [$\overline{1}$00] direction via [100] → [010] → [$\overline{1}$00] process. As both the 90° reversals are simultaneous a single jump in the hysteresis loop was observed. Similar nature of magnetization reversal with a small increase in coercive field (H_*C*_) was observed for sample B along *ϕ* = 0° (Fig. 5(f)). The reason for the increase in H_*C*_ might be due to the strong exchange coupling between Fe and the magnetic C_60_ interface. The domain images for both the samples along *ϕ* = 0° are shown in Fig. S4 in the supplementary information. It should be noted that *ϕ* = 90° is the easy direction for the cubic anisotropy but the hard direction for the uniaxial one (refer to the anisotropy configuration shown in Fig. S3). When applying H_*app*_ along *ϕ* = 90° it leads to two step hysteresis loop as seen in Fig. 5(e). The reason behind the two steps can be explained by the two successive 90° DW motion occurring during the magnetization reversal. The initial 90° DW motion occurs from [010] to [$\overline{1}$00] direction. Just after the completion of this reversal another 90° domain appears by flipping the spin direction from [$\overline{1}$00] to [0$\overline{1}$0]^33^. The Fe/C_60_ bilayer sample follows the similar reversal mechanism (Fig. 5(j)) like the single Fe layer. The domain images corresponding to the *ϕ* = 90° configuration for both the samples A and B are shown in Fig. S6 in the supplementary information.

It should be noted that near the cubic hard axis (45° < *ϕ* < 70°), a major change in the shape of the hysteresis loops has been observed for sample B in comparison to sample A. Therefore, it can be inferred that the presence of magnetic fullerene at the interface between Fe and C_60_ modifies the magnetization reversal. The reason behind this significant change in the hysteresis loop shape around the cubic hard axis for the bilayer sample is the following. The most stable energy configuration for a sample is along its easy axes (*ϕ* = 0° and/or 90°). However, the energy is maximum along the hard axis and the spins can get easily disturbed even with small deviation from the saturation state. Let us first discuss the reversal mechanism in sample A along *ϕ* = 45° (Fig. 5(b)). By decreasing the magnetic field from the saturation state ([110] direction), all the spins try to align along their easy direction [100] via coherent rotation. Considering the anisotropy symmetry schematic shown in Fig. S3, for *ϕ* ≤ 45°, [100] is energetically more favourable as compared to [010]. Referring to Fig. 5(b) we see that while changing the state from the positive remanence by negative magnetic field, it leads to the first reversal. Here the spins get flipped from [100] to [0$$\overline{1}$$0] direction via a 90° DW motion as observed in Fig. S5. Further increase in the field leads to the 2^*nd*^ reversal by switching the spin direction from [0$$\overline{1}$$0] to [$$\overline{1}$$00] through another 90° DW motion (Fig. S5). The later reversal occurs by producing a hump like feature where the intensity is even lower than the negative saturation. It has been reported in previous literature that this asymmetry in hysteresis loops in Fe samples occurs due to the quadratic component of the MOKE. Near the hard axis of the sample the quadratic component becomes dominant over the linear one of the MOKE which gives rise to sudden jumps in hysteresis loops^39–43^. Further in Fig. 5(b) the reversal gets completed with partial rotation of spins from [$$\overline{1}$$00] to [$$\overline{1}$$$$\overline{1}$$0] by applying more negative field. For sample B (Fig. 5(g)), the reversal mechanism for *ϕ* = 45° is similar to sample A (Fig. 5(b)). However, the second 90° reversal is favoured by the magnetic C_60_ layer and the coercivity for the second reversal is less in sample B (Fig. 5(g)) in comparison to sample A.

**Figure 6:**
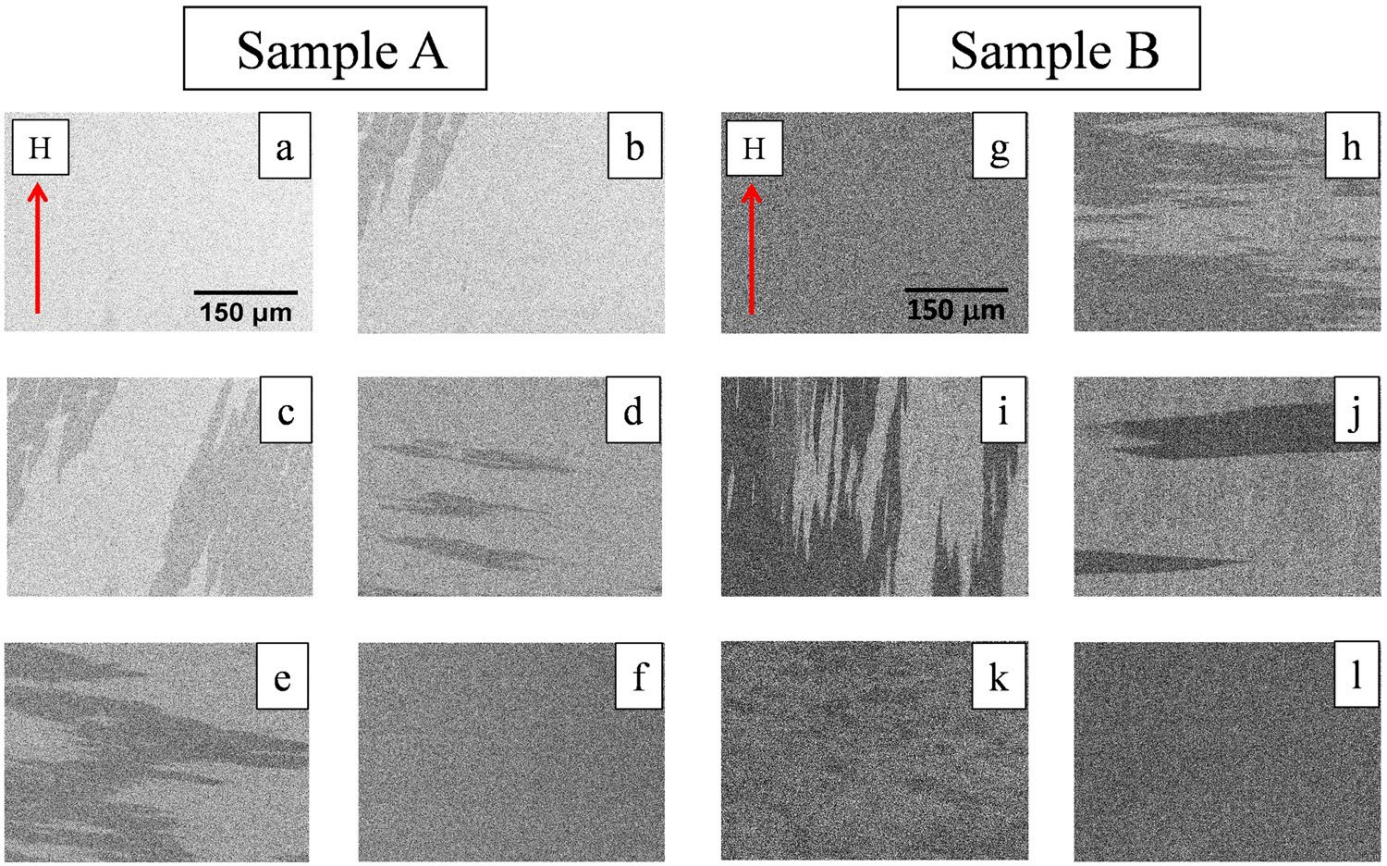
Domain images shown in (**a**–**f**) are for sample A and (**g**–**l**) are for sample B. These domain images were recorded during simultaneous measurement of hysteresis loop along *ϕ* = 50° using Kerr microscopy. The field values for these domain images (**a**–**f**) are shown in the hysteresis loop in Fig. 5(c) marked by 1 to 6, respectively. Similarly, the field values for the domain images (**g**–**l**) are shown in the hysteresis loop in Fig. 5(h) marked by 7 to 12, respectively. All the images for samples A and B are in same length scale shown in (**a**,**g**), respectively.

In the following we discuss the hysteresis loops shown in Fig. 5(c,d,h,i). We have found that *ϕ* ~ 50° is the resultant hard axis of cubic and uniaxial anisotropies for both the samples. Figure 5(c) shows the hysteresis loop for sample A measured along *ϕ* = 50°. Here by decreasing the magnetic field from the positive saturation state (point 1 to 2 in Fig. 5(c)), the spins try to orient themselves via coherent rotation towards [010] direction which is the nearest minimum energy state. Further another reversal occurs from [010] to [$\overline{1}$00] direction via a 90° DW motion (Fig. 6(b,c) corresponding to the points 2 and 3 in Fig. 5(c)). Subsequently, application of magnetic field in negative direction leads to one more reversal from [$\overline{1}$00] to [0$\overline{1}$0] direction via another 90° DW motion (Fig. 6(d,e) corresponding to the points 4 and 5 in Fig. 5(c)). Afterwards, with increase in the field to negative saturation the spins are dragged towards the applied field direction via coherent rotation (Fig. 6(f) corresponding to the point 6 in Fig. 5(c)). For the other branch of the hysteresis loop (negative to positive side i.e. point 6 to 1 in Fig. 5(c)) the sudden increase of the MOKE intensity than the positive saturation one indicates presence of transverse component in the sample. As discussed earlier, this asymmetry (sudden increase) in the hysteresis loop arises due to the dominance of quadratic component of MOKE over the linear one^39–43^. It should be noted that the asymmetry present in the loop along *ϕ* = 45° (Fig. 5(b)) is the reverse of the asymmetry present in the loop for *ϕ* > 45° (Fig. 5(c,d)). This is because the path of the reversal process is just opposite for 40° ≤ *ϕ* ≤ 45° and 45° ≤ *ϕ* ≤ 70°. In case of sample B, the magnetization reversal (Fig. 5(h)) starts with coherent rotation similar to sample A. It should be noted that the effective magnetic field is H_*eff*_ = H_*app*_ + H_*dipolar*_, where, H_*app*_ and H_*dipolar*_ are the external applied field and the dipolar field generated by the Fe underlayer on the interfacial magnetic C_60_ layer, respectively^44^. H_*dipolar*_ is large enough to promote the reversal of the C_60_ layer with less change in the H_*app*_ field. Therefore, even before the switching of the field direction the reversal of the magnetic C_60_ layer was observed (see domain image in Fig. 6(h) corresponding to the point 8 in Fig. 5(h)). Reversal of one layer before the switching of the field direction implies the presence of antiferromagnetic coupling in the sample^45–47^ which is corroborated by the PNR analysis of Fig. 2(b). By further reduction in the field (from point 8 in Fig. 5(h)) first reversal is observed which is from the Fe layer occurring via 90° domain wall motion (i.e. similar to sample A). Immediately afterwards, the second reversal is happening for the Fe layer via another 90° domain wall motion (see domain image in Fig. 6(j) corresponding to the point 10 in Fig. 5(h)). This sudden reversal is due to the strong exchange coupling at the interface between Fe/magnetic C_60_. Upon increase in applied magnetic field in negative direction, the magnetic C_60_ layer undergoes another reversal (domain image shown in Fig. 6(k) corresponding to the point 11 in Fig. 5(h)) to complete the full 180° reversal of the magnetic C_60_ layer. Along *ϕ* = 55° the magnetization reversal mechanism of sample A (Fig. 5(d)) is exactly similar to *ϕ* = 50° configuration. For sample B, the magnetization reversal occurs via two separate 90° DW motion. However, for *ϕ* > 50°, the second 90° reversal is not favoured by the magnetic C_60_ layer and the coercivity for the second reversal increases in sample B (Fig. 5(i)) in comparison to sample A.

## Conclusion

We have studied bilayer of ferromagnet (Fe)/organic semiconductor (Fullerene) and compared the results to the single Fe film. It is found that a thin layer of about 2 nm of C_60_ close to the Fe interface exhibits magnetic moment. The induced moment in C_60_ at the interface is ~1.5 to 3 *μ*_*B*_ per cage. Future theoretical calculation is necessary to elucidate if such high induced magnetic moment in C_60_ is possible via direct charge transfer or any secondary processes are involved. The magnetic C_60_ certainly has profound effect on the magnetization reversal and the domain microstructure when compared to the results obtained on a single layer Fe film. In future systematic study is desired to elucidate the induced magnetism for systems comprising of different magnetic films with variable thickness, crystallinity etc. Our present study shows that non-negligible magnetic moment in the magnetic interface in such FM/OSC bilayers have potential applications in organic spintronics.

## Method

In this article we have studied two samples such as (i) Sample A: MgO (001)/Fe(15 nm)/Ta(3 nm) and (ii) Sample B: MgO (001)/Fe(15 nm)/C_60_(40 nm)/Ta(3 nm). Fe films were grown by DC magnetron sputtering on MgO (001) substrates. C_60_ was deposited by thermal evaporation on top of the Fe layer in sample B. For both the samples the substrate was annealed at T_*ann*_ = 690 °C for an hour prior to deposition. All the samples were deposited at T_*dep*_ = 150 °C. The deposition was performed in a UHV chamber (manufactured by Mantis Deposition Ltd., UK) comprising of both the sputtering and thermal evaporation so that such type of FM/OSC bilayers can be prepared without breaking the vacuum. The base pressure of the deposition chamber was better than 3 × 10^−8^ mbar. The Fe plume in the sputtering chamber was incident at 30° with respect to the surface normal of the substrate. The Fe layers were deposited at a rate of 0.22 *Å*s^−1^, which was monitored by a quartz crystal monitor. To prevent from oxidation, a capping layer of 3 nm thick Ta was deposited on both the samples. ION-TOF’s time of flight secondary ion mass spectroscopy (TOF-SIMS) was performed to analyse the thickness of the individual layers and also to study the nature of their interfaces. A 25 KeV ‘Bi’ ion source was used as the primary ion beam (pulsed) to excite secondary ions. Those secondary ions were analysed by a reflectron based time of flight mass analyser. Another ion source of Cs^+^ (500 eV) was used to sputter out the layers in order to have the depth profile analysis. In order to calibrate the SIMS data, thickness profilometry experiment was performed on the specific hole from where the materials have been dug out during the SIMS measurement. The sputtering area was kept at 300 × 300 *μ*m^2^ and the analysis area was at 100 × 100 *μ*m^2^, respectively. The hysteresis loops along with simultaneous domain images were measured at room temperature by a Magneto optic Kerr effect (MOKE) based microscope in longitudinal mode manufactured by Evico Magnetics Ltd. Germany. MOKE measurements were performed by varying the angle (*ϕ*) between the external magnetic field and the easy axis of the sample at 5° interval. Polarized neutron reflectivity (PNR) was performed on sample B using MARIA reflectometer at FRM II, Garching, Germany at room temperature^48^. A similar sample like sample B with 6 nm Au as capping layer instead of the Ta layer was used for the PNR measurement. The wavelength (*λ*) of the neutrons in the PNR measurements was 6.5 Å. Two scattering cross sections R^++^ (up - up) and R^−−^ (down - down) were measured using a detector depending on the interaction of the neutrons with the magnetic spins in the sample. The first and second signs in the scattering cross section correspond to the polarization of the incident and the reflected neutrons, respectively. A small guiding field of *μ*_0_H ~ 2 mT was applied to maintain the polarization of the incident neutrons. The basic principle of PNR experiment is that one measures the intensity of neutrons as a function of the component of the momentum transfer that is perpendicular to the surface of the magnetic film, i.e. Q_*z*_ = 4*π* sin *θ*/*λ*, where *θ* is the angle of incidence (and reflection) and *λ* is the neutron wavelength. It should be noted that Q_*z*_ is a variable conjugate to the depth z from the surface of the film, therefore performing a scan over a suitable range of Q_*z*_ can provide excellent information on the magnetic depth profile of the magnetic films which may comprise of many independent layers with different magnetic moments. We have performed PNR on sample B by applying magnetic field along *ϕ* ~ 45° (cubic hard axis). The magnetic moment of sample A was obtained by measuring the hysteresis loop at room temperature within ±300 mT magnetic field range using MPMS3 Evercool superconducting quantum interference device (SQUID) manufactured by Quantum Design, USA.

## Electronic supplementary material

Supplementary Information
