# Supplementary material for: Effect of magnetic fullerene on magnetization reversal created at the Fe/C60 interface
Source: Sci Rep. 2018 Apr 3;8:5515. doi: 10.1038/s41598-018-23864-8 (PMC5882892; doi:10.1038/s41598-018-23864-8)
Supplement: Supplementary file 1 — Supplementary Information [file 41598_2018_23864_MOESM1_ESM.pdf]

Supplementary Information

Effect of magnetic fullerene on magnetization reversal created at the Fe/C_{60} interface

Srijani Mallik[1], Stefan Mattauch[2], M. K. Dalai[3,4], Thomas Brückel[2,5], and Subhankar Bedanta[1,*]

[1]Laboratory for Nanomagnetism and Magnetic Materials (LNMM), School of Physical Sciences, National Institute of Science Education and Research (NISER), HBNI, Jatni-752050, India
[2]Jülich Centre for Neutron Science (JCNS), Heinz Maier-Leibnitz Zentrum (MLZ), Forschungszentrum Jülich GmbH, Lichtenbergstr. 1, 85748 Garching, Germany
[3]CSIR - National Physical Laboratory, Dr. K. S. Krishnan Marg, New Delhi - 110012, India
[4]Academy of Scientific and Innovative Research (AcSIR), India
[5]PGI-4: Scattering Methods Forschungszentrum Jülich GmbH 52425 Jülich, Germany
sbedanta@niser.ac.in

In this article we have studied two samples such as (i) Sample A: MgO (001)/Fe(15 nm)/Ta(3 nm) and (ii) Sample B: MgO (001)/Fe(15 nm)/C_{60}(40 nm)/Ta(3 nm).

Polarized neutron reflectivity

The fit for the polarized neutron reflectivity (PNR) data of sample B at saturation (Figure 2(a)) yeilds an induction of 2.95 μ_B magnetic moment per C_{60} cage at the interface. If we consider that the induced moment in C_{60} is due to the direct charge transfer of 3 electrons from Fe then the potential step at the interface becomes high (as per the below calculation). The potential can be found out by using Helmohltz equation i.e.
$\Delta V = \mu \times Cos\phi/(\varepsilon_r \times \varepsilon_0)$
where, μ is the dipole moment per unit area, $\phi \simeq 0$ is the angle between dipole and surface normal, $\varepsilon_r \sim 4.2$ (typical value for C_{60} is between 4 to 4.5),[1] and ε_0 is the permittivity of vacuum. If we assume the area of interface which probably is directly interacting with the magnetic C_{60} to be $\sim$ 1.3 nm^2 (see figure S1 for clarification) and the distance between point charges at C_{60} and Fe sites is about 0.2 nm near interface, then with a charge transfer of 3 electron per C_{60},
$\mu = (3 \times 0.2)/1.3$ e/nm = 0.462 e/nm
So, $\Delta V = (0.462 \times 1.6 \times 10^{-19} \times 10^{9}) /(4.2 \times 8.85 \times 10^{-12})$ V = 1.99 V

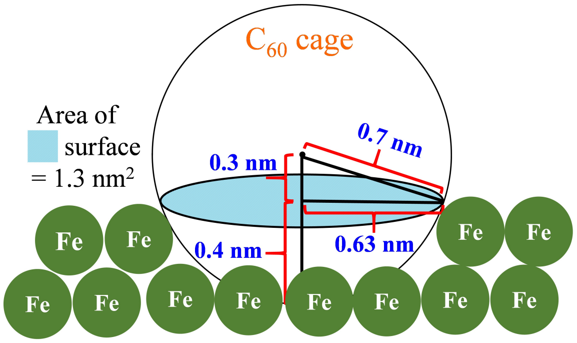

Figure S1. Schematic of one C_{60} cage sitting on top of the Fe layer to calculate the area of contact between C_{60} and Fe surface.

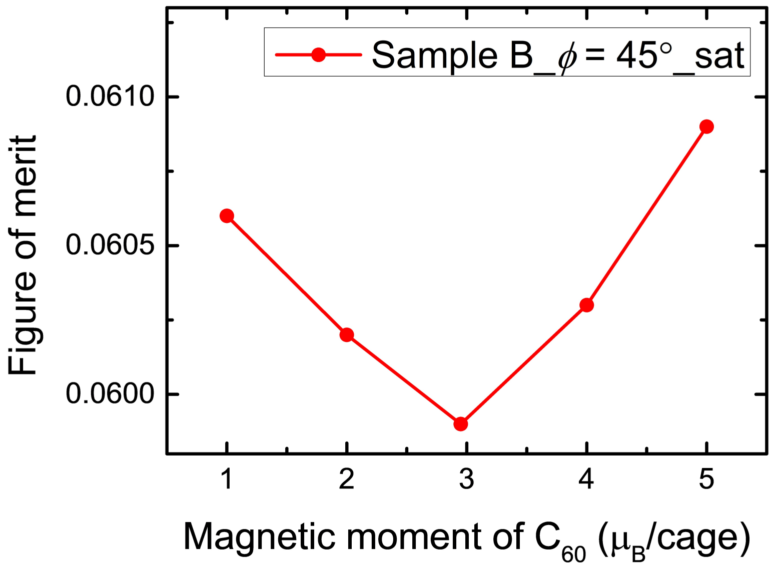

Figure S2. Figure of merit (FOM) versus different values of induced magnetic moment of C_{60} to show the best fitted value of the induced moment.

Therefore, by considering 3 electron transfer yields high potential step at the interface which is unrealistic. In this context we have tried to fit the PNR data with both lower as well as higher magnetic moments with respect to 2.95μ_B of C_{60}. It was observed that the quality of the fits does not change much considering different values (1 to 5 μ_B) of C_{60} induced moments. However, the best fit has been chosen on the basis of the 'figure of merit$'$ which is minimum for the C_{60} magnetic moment $\sim$ 2.95 μ_B/cage (Figure S2).

MOKE magnetometry

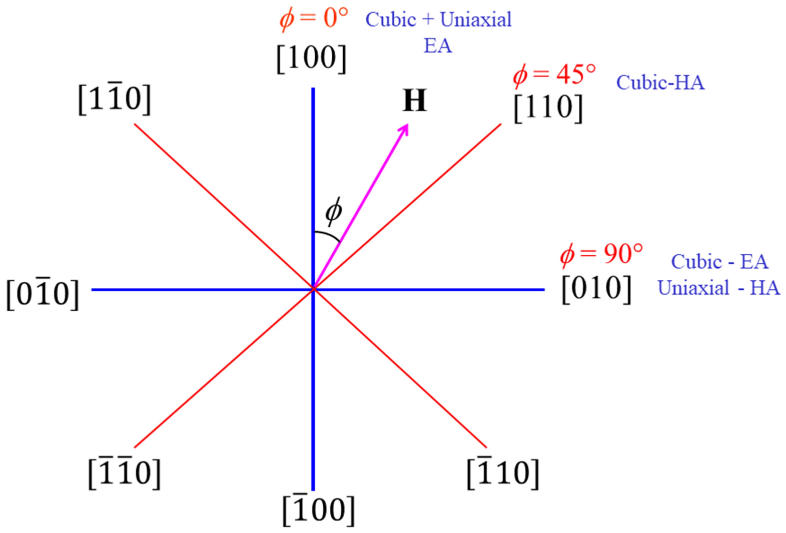

Figure S3. Schematic for anisotropy configuration of sample A and B

The anisotropy symmetry of both the samples A and B is depicted in figure S3. Due to the epitaxial growth of Fe on MgO (001) substrate, cubic anisotropy is present in the samples. The Fe layer was deposited under oblique angle of incidence (the angle between the substrate normal and the Fe plume was fixed at 30°). Because of the oblique angular deposition, uniaxial anisotropy is induced in these samples. Therefore, two types of anisotropies (cubic + uniaxial) are present in our samples

where the easy axis of the cubic anisotropy is superimposed with the easy direction of the uniaxial anisotropy. To check the reproducibility of the data, we have prepared multiple samples at different times with similar growth parameters. It has been observed from the MOKE measurement that the nature of the hysteresis loops remain similar for all the samples.

Kerr microscopy

Figure S4, S5 and S6 show the hysteresis loops and their corresponding images for samples A and B along 0°, 45° and 90°, respectively. Along the cubic easy axes i.e. $\phi = 0°$ and 90°, the magnetization reversal is governed by two simultaneous (Figure S4(a) - (e)) or successive (Figure S6(a) - (e)) 90° domain wall motion, respectively. The effect of magnetic C_{60} layer on the magnetization reversal of Fe is not prominent along the easy axes. Therefore, along $\phi = 0°$ and 90°, the magnetization reversal mechanism for sample B (Figure 4(f) - (j) and Figure S6(f) - (j)) exhibits similar behavior that of sample A. Along the cubic hard axis i.e. $\phi = 45°$, the magnetization reversal for sample A is controlled by a combination of two 90° domain wall motion and partial rotation (Figure S5(a) - (e)). For sample B, the reversal mechanism is similar, however, the distinct plateau region between two 90° reversal observed in case of sample A is absent for sample B (Figure S5(f) - (j)). It can be inferred that the magnetic C_{60} layer is helping to generate the 2nd 90° domain which leads to successive motion of two such domains.

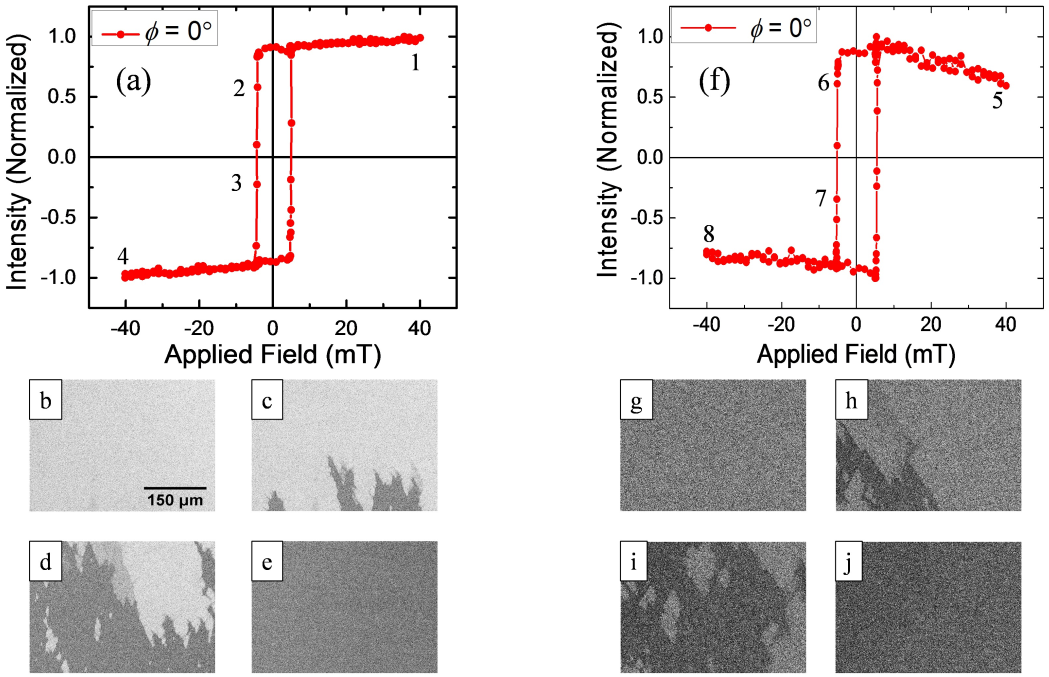

Figure S4. Hysteresis loops and corresponding domain images for (a) sample A and (f) sample B along $\phi = 0°$ measured using Kerr microscopy. The domain images shown in (b - e) and (g - j) correspond to the points (1 - 4) and (5 - 8) in the hysteresis loops (a) and (f), respectively. All the images are in same length scale given in the domain image (b).

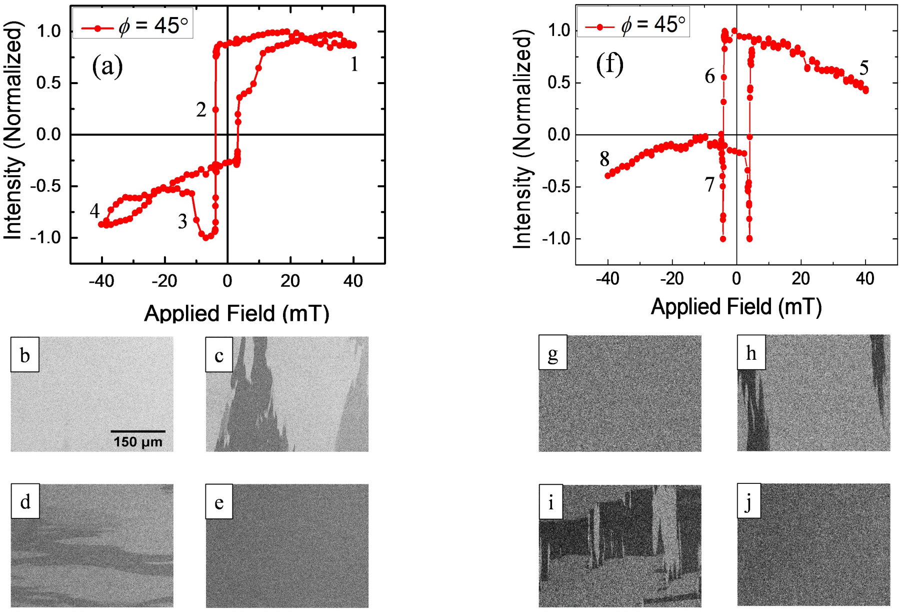

Figure S5. Hysteresis loops and corresponding domain images for (a) sample A and (f) sample B along $\phi = 45°$ measured using Kerr microscopy. The domain images shown in (b - e) and (g - j) correspond to the points (1 - 4) and (5 - 8) in the hysteresis loops (a) and (f), respectively. All the images are in same length scale given in the domain image (b).

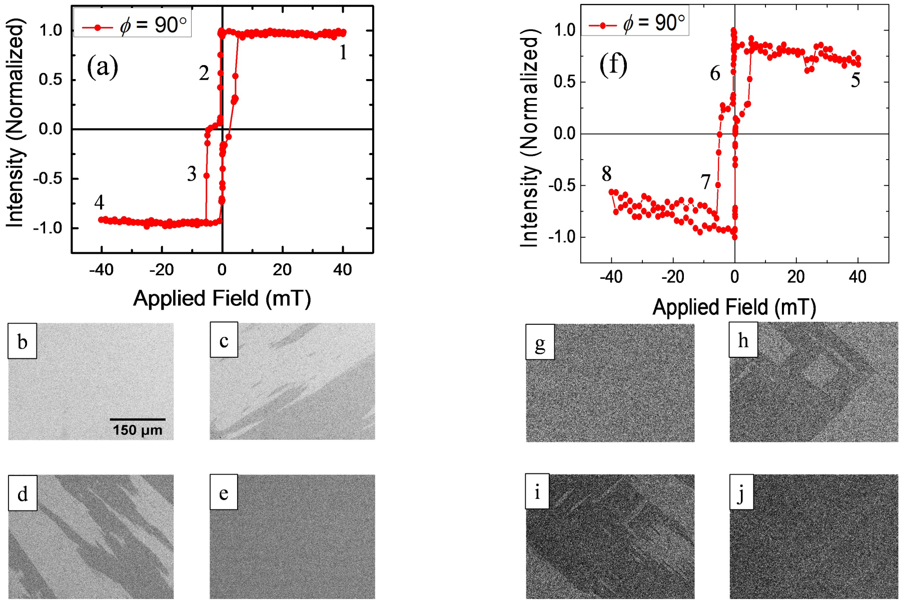

Figure S6. Hysteresis loops and corresponding domain images for (a) sample A and (f) sample B along $\phi = 90°$ measured using Kerr microscopy. The domain images shown in (b - e) and (g - j) correspond to the points (1 - 4) and (5 - 8) in the hysteresis loops (a) and (f), respectively. All the images are in same length scale given in the domain image (b).

References

1. Hansen, P. L., Fallon, P. J., & Kratachmer, W. An EELS Study of Fullerite - C_{60}/C_{70} *Chem. Phys. Lett.* **181**, 367 (1991).
